# Dissecting Pathogenetic Mechanisms and Therapeutic Strategies in *Drosophila* Models of Myotonic Dystrophy Type 1

**DOI:** 10.3390/ijms19124104

**Published:** 2018-12-18

**Authors:** Anissa Souidi, Monika Zmojdzian, Krzysztof Jagla

**Affiliations:** GReD, INSERM U1103, CNRS, UMR6293, University of Clermont Auvergne, 28 Place Henri Dunant, 63000 Clermont-Ferrand, France; anissa.souidi@uca.fr (A.S.); monika.zmojdzian@uca.fr (M.Z.)

**Keywords:** DM1, muscular dystrophy, animal model, *Drosophila*, drug screen, genetic screen, therapeutic targets

## Abstract

Myotonic dystrophy type 1 (DM1), the most common cause of adult-onset muscular dystrophy, is autosomal dominant, multisystemic disease with characteristic symptoms including myotonia, heart defects, cataracts and testicular atrophy. DM1 disease is being successfully modelled in *Drosophila* allowing to identify and validate new pathogenic mechanisms and potential therapeutic strategies. Here we provide an overview of insights gained from fruit fly DM1 models, either: (i) fundamental with particular focus on newly identified gene deregulations and their link with DM1 symptoms; or (ii) applied via genetic modifiers and drug screens to identify promising therapeutic targets.

## 1. Introduction

Myotonic dystrophy type 1 (DM1, OMIM #160900), also called Steinert myotonic dystrophy, is the most common muscular dystrophy in adults, with a worldwide incidence of 1/8000 [1]. DM1 is an autosomal dominant multisystemic disease with characteristic symptoms including myotonia (inability of muscles to relax after contraction), muscular dystrophy with progressive muscle weakness, heart defects, cataracts and testicular atrophy. The genetic basis for DM1 is an expanded CTG repeat in the 3′-untranslated region of the *dystrophia myotonica protein kinase* (*DMPK*) gene, which maps to 19q13.3 [2] and encodes a putative serine/threonine protein kinase. Healthy individuals have between 5 and 37 CTG repeats, whereas the DM1-affected population carries expansions from 50 up to 4000 CTGs [1]. Longer repeat lengths correlate with early onset and increased severity of the disease [3]. There are four main categories of DM1 phenotypes dependent of CTG repeat size: congenital, childhood-onset, adult-onset and late-onset/asymptomatic [3]. Congenital form (CDM1) with more than 1000 CTG repeats, is characterized by severe neonatal hypotonia, intellectual disability and facial diplegia that appear from birth [4]. Cardiac involvement with conduction abnormalities and arrhythmia, as well as respiratory failure, are common in CDM1 and are often fatal [5]. Childhood-onset form (50–1000 CTG repeats) appear between 1 and 10 years and involves weakness in muscles of lower legs, hands, neck, face and myotonia of muscles of the tongue and forearm. Conduction defects are also commonly involved. Finally, adult onset DM1 is apparent between 10–20 years, leading to muscle weakness, myotonia, cataracts, respiratory disturbance but also to a large set of cardiac involvements including conduction abnormality, mitral valve prolapse [6] but also alterations in ventricular diastolic function [7], abnormal ventricular late potentials [8] and arrhythmias that are positively correlated with size of CTGs [9]. In addition, DM1 patients with missplicing of *insulin receptor* (*IR*) gene present insulin resistance and mild type-2 diabetes [10]. Late onset DM1 is detected in 30–70 years old patients carrying 50–100 CTGs and is characterized by mild myotonia and cataracts whereas individuals with 38–49 CTG repeats, are in general asymptomatic.

In DM1 patients, CTG repeats are unstable and show size variation in different tissue and cell types causing somatic mosaicism. In post-mortem tissues from an adult patient, (CTG)n expansions in brain, skeletal muscle, cardiac muscle, testes and liver were all greater than in leukocytes [11]. In addition, the size of the CTG repeats appears to increase over time in the same individual and across generations, with increasing severity of the disease. Children may thus inherit considerably longer repeat lengths than those initially present in the transmitting parent. This phenomenon is known as genetic anticipation, in which disease severity increases and/or age of onset of disease decreases from one generation to the next [12]. However, CTG repeat size does not always increase in successive generations of DM1 families. Intergenerational contraction of CTG repeats also occurs in about 6.4% of transmissions, most frequently in paternal transmission (10%) [13].

To characterize molecular defects underlying DM1 pathogenesis, different vertebrate and invertebrate animal models have been successfully generated. Interestingly, *Drosophila* has been shown to mimic DM1 phenotypes. This review describes how the simple *Drosophila* model has helped us understand the complex molecular mechanisms underlying DM1 and test and identify therapeutic strategies to ameliorate the DM1 phenotype. 

## 2. DM1 Pathogenesis

Essentially three models have been proposed to account for the genetic inheritance and molecular characteristics of DM1. However, given the complexity of the disorder, all three mechanisms may contribute to the DM1 phenotype, as explained below. 

According to the first DM1 pathogenesis model, CTG expansion affects the level of *DMPK* expression in cis by altering its transcription or by the retention of CUG expanded transcripts, which may lead to haploinsufficiency. Decreased levels of the *DMPK* mRNA and protein were found associated with the adult form of DM1 [14]. However, *DMPK−/−* mutant mice show only minor size changes in head and neck muscle fibres at older age and do not develop other DM1 symptoms including the fibre-type dependent atrophy, myotonia, cataract and mal-infertility. Reduced DMPK expression is therefore not the only condition for development of DM1 [15].

In the second model, the CTG triplet expansion may alter chromatin structure and induce repression of *DMPK* neighbouring genes. The CTG expansion in the *DMPK* 3′UTR is located immediately upstream of the *SIX homeobox 5* (*SIX5*) promoter region and was shown to lower SIX5 expression [16]. The *SIX5* gene encodes a homeodomain transcription factor involved in distal limb muscle development in mice [17] and its *Drosophila* ortholog is essential for eye development in the fly. *Six5−/−* mutant mice developed ocular cataracts and infertility but no apparent abnormalities of skeletal muscle function and failed to reproduce most of the symptoms of DM1 patients [18].

Finally, in the third model, repeat expansions, once transcribed into RNA, exert a gain-of function toxic effect in the cells. This hypothesis is supported by the fact that the transgenic mice expressing expanded, noncoding CUG repeats under the control of the human skeletal actin promoter develop myotonia, a classical DM1 feature. Muscle histology of these mice also shows central nuclei, ringed muscle fibres and variability in fibre size similar to the histological features observed in DM1 patients. Thus toxic CTG repeats could cause DM1 phenotypes independently of reduced levels of DMPK [19]. Pathogenic features of transcripts carrying expanded CUG repeats rely on the formation of secondary structures with a hairpin shape that are retained in nuclear foci observed in both cultured cells [20] and in biopsy tissues from DM1 patients [21]. These nuclear foci sequester RNA-binding proteins such as muscleblind-like 1 (MBNL1) [22]. Consequently, the activity of MBNL1 as a splicing regulator is impaired, resulting in aberrant alternative splicing of its target genes [23]. Double-stranded RNA structures also abnormally activate the RNA-dependent protein kinase R (PKR), which hyper-phosphorylates another splicing factor, CUGBP Elav-Like Factor 1 (CELF1) resulting in its stabilization and increased splicing activity in DM1 skeletal muscle and heart tissues [24].

Interestingly, MBNL1 and CELF1 play antagonistic roles. During development, the CELF proteins promote the inclusion of specific foetal exons in embryonic and neonatal tissues, whereas postnatal activation of MBNL leads to foetal exon skipping and expression of adult protein isoforms [25]. To ensure these specific functions, during heart development, CELF proteins are down-regulated more than 10-fold and MBNL1 protein is concomitantly up-regulated nearly 4-fold. This MBNL1/CELF1 balance is then reversed in adulthood. Using transgenic mice, it was demonstrated that reproducing the embryonic expression patterns for CELF1 and MBNL1 in adult heart induced the embryonic splicing patterns for more than half of the developmentally regulated alternative splicing transitions [26]. Remarkably, such a reversed, embryonic-like MBNL1/CELF1 ratio is found in the DM1 context, leading to the mis-splicing and abnormal expression of foetal isoforms of several genes in adult tissues.

The important role that MBNL1 plays in DM1 is supported by the Mbnl1 knockout mice phenotypes, which show several DM1 features including misregulated mRNA splicing, histopathological muscle changes, cataracts and myotonia [27]. Consistently, overexpression of Mbnl1 in skeletal muscle of the poly(CUG) mouse DM1 model rescues the myotonia phenotype concurrently with a restoration of the normal adult-splicing patterns [28]. The capacity of Mbnl1 to rescue the main DM1 symptoms is also observed in the *Drosophila* DM1 model, where cardiac overexpression of Muscleblind (Mbl), the *Drosophila* Mbnl1 ortholog, is sufficient to rescue the heart dysfunctions and reduced survival of DM1 flies [29]. 

The RNA-binding factor CELF1 is another key component in the development of the DM1 phenotype. CELF1 protein localizes mainly to the nuclei where it acts as a splice regulator but can also be detected in the cytoplasm. It binds to the GU-rich element (GRE) and mediates GRE-dependent mRNA decay, which regulates the expression of a large subset of human transcripts [30]. CELF1 can also act as a deadenylation factor. It has been suggested that in the DM1 context, the expanded CUG repeats can affect the activity of CELF1, leading to a trans-dominant effect on RNA processing [31]. It has also been demonstrated that unlike MBNL1, the CELF1 down-regulation was not sufficient to rescue mis-splicing in the DM1 mouse model, although deterioration of muscle function was prevented and muscle histopathology improved [32].

## 3. *Drosophila* Could Serve as a Model Organism for DM1

*Drosophila* melanogaster has long been recognized as one of the most powerful genetic systems for analysing the function of human disease genes. Comparison of human genes associated with at least one mutant allele in the Online Mendelian Inheritance in Man (OMIM) database against the genome sequence of D. melanogaster revealed that 714 distinct human disease genes (77% of disease genes searched for) matched 548 unique *Drosophila* sequences [33]. Furthermore, sequencing of the *Drosophila* and the human genomes revealed remarkably high similarities between the fly and humans [34]. Most importantly, molecular pathways required for the development and cell biology have been highly conserved since the evolutionary divergence of flies and humans. This finding has made *Drosophila* a model system well-suited to addressing molecular mechanisms of human pathologies including those, like DM1, that affect skeletal and cardiac muscles.

Several fly models of DM1 have been generated and applied to unravel mechanisms underlying expanded CUG repeat toxicity. The first model consisted of expressing 11, 48, 56 or 162 pure CTG repeats in the context of the 3′UTR of a *Green Fluorescent Protein* (*GFP*) reporter gene. Only in muscle cells expressing 162 CTGs were discrete ribonuclear foci co-localizing with Mbl detected without obvious locomotor activity perturbation, muscle defects or reduced lifespan of the animals [35], suggesting that repeat expansion was insufficient. Shortly afterwards, De Haro and co-workers examined the effect of an increased repeat number using 480 interrupted CTG repeats expressed in adult muscles or the eye. *Drosophila* DM1 models developed age-dependent degenerative phenotypes in muscle or eye tissue and showed accumulation of repeat carrying transcripts in nuclear foci co-localizing with Mbl, like in muscles of DM1 patients [36]. Similar transgenic flies, expressing 480 interrupted repeats, were generated by Garcia-Lopez and co-workers. They demonstrated that CUG_480_-expressing flies reproduced, additional to degenerative phenotypes, a splicing misregulation and central nervous system alterations. Interestingly, the degenerative phenotype was dependent on the CUG tract length [37]. Recently, a series of fly models was generated with a non-coding, uninterrupted CTG repeat expansion of 19, 130, 200, 230, 250 and 270 in length into the 3′UTR of the *DsRed* gene. As demonstrated in previous study, the CTG-toxicity was detected in flies expressing 200 repeats or more, suggesting that the severity of phenotypes in *Drosophila* DM1 models is positively correlated with the size of the CTG repeats, similar to what has been observed in DM1 patients. Interestingly, the co-expression of CTG with expanded CAG repeat transcripts leading to generation of triplet repeat-derived siRNAs that enhance CTG toxicity [38].

As DM1 affects several organs including the heart, *Drosophila* has also been applied to generate a cardiac DM1 model. As evidence that *Drosophila* could be used for modelling human heart disorders, Cammarato and co-workers report that 73% of fly survival genes have human and/or mouse orthologs with critical heart functions and that 40% of them are associated with cardiac disorders including cardiomyopathy, myocardial infarct, cardiac arrest and heart failure. For example, *Cathepsin B1* (*CtsB1*) gene is conserved between *Drosophila* and human (*cathepsin B*) and is associated to cardiac arrest [39].

The heart of *Drosophila* (or dorsal vessel) is formed, like in vertebrates, during early stages of embryogenesis from cardiac mesoderm. Importantly, several factors necessary for cardiac development in humans such as *Nkx2.5* and *Hand* have *Drosophila* orthologs (*Tinman, Tin*) [40], (*dHand*) [41] exerting conserved cardiogenic functions. Moreover, the main signalling pathways and genes ensuring cardiac function are also highly conserved [42] and operating in the *Drosophila* heart. As oxygen distribution in *Drosophila* is heart-independent and ensured by tracheal system, the fruit fly heart represents an attractive model system for studying severe pathological conditions such as cardiac arrest, which does not led to death in the fly.

The main cardiac dysfunctions associated with DM1 correspond to conduction defects [43] with potentially fatal ventricular and/or atrial arrhythmias [44] and mechanical diastolic and/or systolic dysfunction that can lead to combined systolic and diastolic heart failure [45]. Importantly, *Drosophila* DM1 models have reproduced all these cardiac disorders [46,47].

In addition to DM1, *Drosophila* has also been used to better understand molecular pathways associated with myotonic dystrophy type 2 (DM2), the second type of myotonic dystrophy. Similar to DM1, DM2 is caused by unstable, noncoding repeat expansions (CCTG)_n_ in *CNBP* gene encoding a CCHC-type zinc finger protein. The CCTG repeat containing transcripts, like those carrying CTG repeats, sequester RNA biding proteins including MBNL1. Recently, Yenigun and co-workers generated transgenic flies expressing 106 CCUG repeats in muscle. These flies exhibit RNA foci formation and aberrant splicing of MBNL1-dependant transcripts such as *Fhos* and *TNNT2* [48] making them well adapted for studying DM2.

## 4. Identification of New Mechanisms Underlying DM1 Using *Drosophila* Models

Several splicing defaults and transcriptional alterations have been described in DM1 patients and in different DM1 models including *Drosophila*. In adult DM1 flies expressing 480 CTGs, among genes involved in muscle development *Z band alternatively spliced PDZ-motif protein 52* (*Zasp52*), ortholog of *LDB3* in human, encoding a Z-band component was found aberrantly spliced [37] and could contribute to the disorganization of the sarcomere and Z-band disruption, also reported in DM1 patients [38]. In addition to *Zasp52*, the *troponin T* encoding another sarcomeric protein that controls the calcium-mediated interaction between actin and myosin [49] as also mis-spliced in the *Drosophila* DM1 model [37]. The aberrant splicing of *troponin T* ortholog *cardiac troponin T* (*cTNT*) is detected in DM1 patients [50] and thus appears to be an evolutionarily conserved mis-splicing event underlying DM1 pathogenesis. Interestingly, expression of abnormal splice isoforms of sarcomere components in DM1 context is consistent with the affected sarcomeric apparatus in partially paralyzed Mbl mutant larvae [51], suggesting that Mbl could be responsible for these mis-splicing events. The alternative splicing defects are not the only mechanisms underlying DM1-associated alterations of gene expression, it was demonstrated that nuclear accumulation of toxic CUG repeats could also affect gene expression independently of splicing [52]. In this study performed in our group, several new inducible *Drosophila* DM1 lines with an increasing number of noncoding CTG repeats (240, 480, 600, 960) were generated and analysed for their pathogenic potential in larval somatic muscles [52]. Data generated from this model confirmed that the *Drosophila* larva could be used for assessing DM1 phenotypes and underlying gene deregulations. In addition to nuclear foci formation and Mbl sequestration, the DM1 larval muscles recapitulated the major DM1 symptoms in a repeat-size dependent manner. These phenotypes included muscle hypercontraction, splitting of muscle fibres, reduced fibre size or myoblast fusion defects [52].

**Figure 1:**
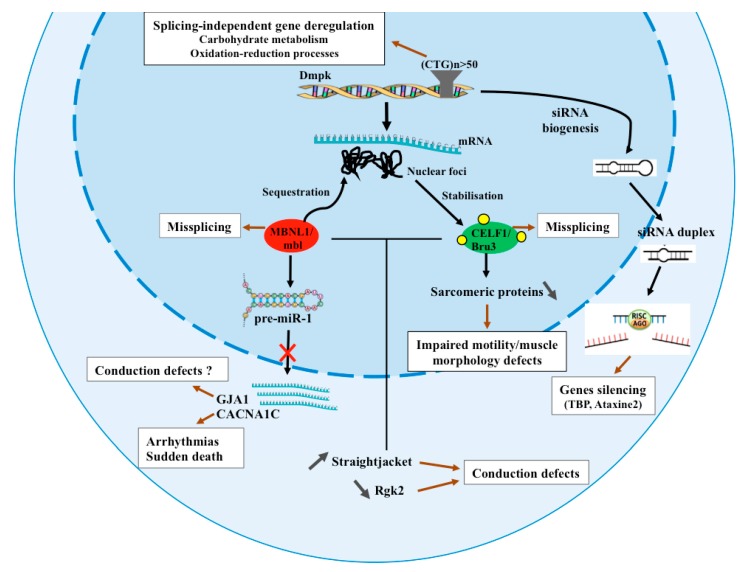
Molecular mechanisms underlying DM1 pathology identified in *Drosophila*. DM1 is caused by microsatellite repeat expansions (*n* > 50 CTG repeats) in 3’UTR region of *DMPK* gene. Mutant *DMPK* transcripts are retained in the nucleus and form nuclear foci that sequester MBNL1/Mbl and stabilize CELF1/Bru3 proteins leading to missplicing. Bru3 stabilization also induces down-regulation of sarcomeric proteins associated with impaired motility and muscle morphology defects [59], whereas loss of MBNL1/Mbl blocks *pre miR-1* processing [53] leading to up regulation of its target genes such as *GJA1* and *CACNA1C* suggested as associated with conduction defects and arrhythmias/sudden death, respectively [45]. Both Bru3 stabilization and Mbl sequestration induce up regulation of *Stj* and down regulation of *Rgk2* expression associated with conduction defects [47]. CTG repeats could be transcribed in both directions leading to CAGn–CUGn double stranded complexes and formation of siRNA duplex that interact with RISC complex to target the expression of genes containing CAG repeats, such as *Ataxin-2* (*ATXN2*) and *TATA binding protein* (*TBP*) [38]. Long CTG repeats induce down regulation of genes in a splicing independent manner including genes involved in carbohydrate metabolism and oxidation-reduction processes [52].

Using this model, several splice-independent deregulated genes were identified. Among candidates specifically down-regulated by CTG repeats are genes involved in metabolic processes (Figure 1), in particular in carbohydrate metabolism *amylase distal* (*Amy-d*), *amylase proximal* (*Amy-p*), *CG32444*, *CG9466* and *CG9468* and oxidation-reduction processes (*Cyp6a18*, *Cyp6w1*, *Cyp304a1* and *CG2065*) [52]. However, the link between deregulation of these genes and the DM1 phenotype remains to be determined. 

Other mechanisms have been described as associated with DM1 phenotypes. These include the formation of siRNA [38] and microRNA (miRNA) deregulation [53]. The *DMPK* gene displays bi-directional transcription, generating anti-sense CAG repeat transcripts in addition to the CTG transcripts. It was shown that the co-expression of CUG together with CAG bearing repeat transcripts induced the enhancement of CTG-toxicity in the fly and was due to the biogenesis of small RNAs. These siRNAs targeted the expression of genes such as *Ataxin-2* (*ATXN2*) and *TATA binding protein* (*TBP*), which bear long CAG repeats in both the fly and humans (Figure 1) [38].

The involvement of miRNAs in DM1 is well studied. Based on biopsy analysis from DM1 patient samples, numerous miRNAs, conserved between *Drosophila* and human, were found deregulated. These include *miR-1*, *miR-7* and *miR-10*, which are down-regulated in both DM1 *Drosophila* models and in DM1 patients. Interestingly, Garcia-Lopez and co-workers demonstrated that the reduced level of *miR-1* in DM1 hearts was due to a lower MBNL1 level and its incapacity to drive *miR-1* maturation [53]. In physiological context MBNL1 binds to a UGC motif located within the loop of *pre-miR-1* and competes for the binding with LIN28. LIN28 promotes *pre-miR-1* uridylation by terminal uridylyltransferase 4 (TUT4) and blocks Dicer processing. However, in DM1 context, MBNL1 is sequestered by CUG repeats in nuclear foci, this enables LIN28 to bind into *pre-miR-1* leading to it uridylation down-regulate it processing. As a consequence, reduction of *miR-1* levels leads to an increased expression of *gap junction protein alpha 1* (*GJA1*) and *calcium voltage-gated channel subunit alpha1 C* (*CACNA1C*) in DM1 affected hearts [54].

*CACNA1C* encodes the main calcium channel in heart tissue and its gain-of-function mutations result in arrhythmias and sudden death [55]. *GJA1* encodes the gap-junction channels in the heart and is responsible for intracardiomyocyte conductance. This finding suggests that CACNA1C and GJA1 up-regulation may contribute to the cardiac dysfunctions observed in DM1 patients (Figure 1). The reduced level of *miR-7* is the result of the down-regulation of its pre-miRNA precursor [53]. The link between *miR-7* and DM1 remains to be addressed, but the fact that *miR-7* was down-regulated in patients with end-stage dilated cardiomyopathy (DCM) [56] suggests it could contribute to cardiac DM1 defects.

In addition, it was found that *miR-7a/b* down-regulation is associated to myocardial infarction (MI), injuries and cardiomyocyte apoptosis. Thus, this down regulation promotes overexpression of important genes that are involved in cell apoptosis such as *poly*(*ADP-ribose*) *polymerase* (*PARP*) [57] and *specificity protein 1* (*SP1*) [58]. 

MBNL1 sequestration and CELF1 accumulation were the first and now the most thoroughly studied mechanisms of DM1 pathogenesis, but the specific contribution of CELF1 to DM1-associated phenotypes has not yet been entirely elucidated. Recent work by our group [59] using *Drosophila* as a model revealed new functions of the fly CELF1 counterpart, Bruno-3 (Bru-3) and its contribution to the DM1 phenotype. Our data demonstrate that the increased level of Bru-3 in muscles contribute to impaired larva motility and muscle morphology defects in DM1. Genome-wide transcriptional profiling of Bru-3 overexpressing larvae has led to identification of a set of 11 genes encoding conserved sarcomeric components whose expression is down-regulated (Figure 1). These include *α-Actinin* (*Actn*), *Myosin heavy and light chains* (*Mhc*, *Mlc1* and *Mlc2*), *Tropomyosin 1* and *2* (*Tm1* and *Tm2*), *Troponin I* (*wupA*) and *C47D* (*TpnC47D*), *bent*, *Paramyosin* (*Prm*), *Zasp52* and *Unc-89* [59]. Interestingly, as demonstrated for *Actn*, the down-regulation of sarcomeric genes involves the cytoplasmic Bru-3 function and its potential role in co-translational mRNA decay [59]. 

Another recent study reporting transcriptional profiling of the cardiac DM1 models [59] revealed that the inhibition of mbl or overexpression of Bru-3 specifically in the fly heart, that mimic the pathogenic MBNL1/CELF1 misbalance in DM1 patient, induce asynchronous heartbeat and cardiac arrhythmia (Figure 1). The data analysis revealed that the cardiac disorders observed in these DM1 models are the result of deregulation of genes involved in cellular calcium level and cardiac conduction. Among them are: *inactivation no afterpotential D* (*inaD*), *syntrophin-like 1* (*syn1*), *Rad*, *Gem/Kir family member 2* (*Rgk2*) and *straightjacket* (*stj*), all conserved in humans [47]. In particular, increased expression of *stj*, ortholog of *CACNA2D3* in human, which encodes a regulatory subunit of a voltage-gated calcium channel CACNA1C/Cav1.2 was found to influence cardiac contractions. Up-regulation of stj leads to increased Ca^2+^ inward current, which induces asynchronous heart beating and mimics conduction defect phenotypes observed in DM1 contexts. Interestingly, reducing the expression of stj in cardiomyocytes of DM1 flies ameliorates asynchronous heart beating [47] strongly suggesting a novel pathogenic mechanism underlying conduction disturbances in DM1. This is supported by the overexpression of CACNA2D3 observed in human cardiac tissue from DM1 patients [47].

## 5. Discovering Potential Gene and Drug-Based Therapeutic Strategies Using Fly DM1 Models

In addition to its role in dissecting molecular mechanisms underlying DM1 pathogenesis, the *Drosophila* model also appears well-suited to testing gene- and drug-based therapeutic strategies. Garcia-Lopez and co-workers performed a genetic screen in their fly DM1 model to identify genetic modifiers of the rough eye phenotype, generated by eye-targeted expression of toxic CUG repeats (480 interrupted CTGs). This led to the identification of six suppressor: *cap-n-collar* (*cnc*), *Nucleosome remodelling factor-38kD* (*Nurf-38*), *fear-of-intimacy* (*foi*), *Coronins* (*coro*), *C-Src Kinase* (*csk*) and *spinster* (*spin*); and three enhancer genes: *seven up* (*svp*), *Viking* (*vkg*) and *CG4589* (Table 1) [37] of CTG-toxicity.

The capacity of these genes to modify DM1 phenotypes remain to be validated by testing their effects into DM1 vertebrate models. Another screen for suppressors of CUG-induced myopathy [60] identified smaug (smg), which was shown to prevent muscle wasting and restore muscle function when overexpressed in *Drosophila* (Table 1). Interestingly, increased levels of human SMAUG1 have an ability to correct the abnormally high nuclear accumulation of CELF1 in myoblasts from DM1 patients and restore its translational activity [60].

Aberrantly activated apoptosis and autophagy pathways appear to be involved in muscle-loss phenotype in DM1 [61]. Genes that negatively regulate apoptosis and autophagy are down-regulated in skeletal muscle biopsies from DM1 patients, whereas autophagy-related genes such as *Atg4*, *Atg7* and *Atg12* were significantly up-regulated in fly muscles expressing CTG repeats [61], similar to what has been observed in DM2 *Drosophila* model expressing CCUG [62]. Based on these findings, Bargiela and co-workers tested the effects of inhibition of apoptosis or autophagy pathways on DM1 flies expressing 480 interrupted CTG repeats. This fly model presented muscle size reduction associated to muscle atrophy and wasting. These phenotypes were rescued by overexpression of *Drosophila* inhibitor of apoptosis 1 (DIAP1) or by reducing autophagy via overexpression of mbl and mTOR. Silencing of autophagy regulatory genes also led to rescue the muscle loss phenotype [61].

Cerro-Herreros and co-workers applied another strategy dedicated to boost Mbl expression in the DM1 context by using sponge constructs against *dme-miR-277* and *dme-miR-304.* These two *Drosophila* miRNAs negatively regulate Mbl transcript levels, so that their silencing allowed an increased Mbl expression. The inhibition *dme-miR-277* led to reduced muscle atrophy, rescued motor function and extended the lifespan of DM1 flies. In addition, the inhibition of *dme-miR-304* rescues the missplicing of *CyP6W1*, *Fhos* and *Serca1* transcripts [63].

In addition to genetic modifiers, *in vitro* and *in vivo* efforts using chemical compounds have been tested to rescue DM1 phenotypes. Attempts have been made to target each step of the pathogenesis, but the most promising therapeutic strategies have been focused on bioactive molecules that bind to the toxic RNA target preventing its interaction with MBNL1 protein. Distinct small molecules have been found to impair the MBNL1-CUGexp complex and thus improve DM1 phenotypes [37,64,65]. In *Drosophil*a, initial drug screening performed by Garcia-Lopez et al. (2008) led to the selection of ten drugs acting as suppressors of CUG-mediated neuronal toxicity. Most of these substances are inhibitors of neuronal excitation, monoamine uptake or substances that affect sodium and calcium metabolism (Table 1) [37]. Another screen was designed in order to develop therapeutic strategy that reduce the CUG-RNA hairpin formation and rescue a semi-lethal pupal phenotype induced by brain-targeted expression of 480 CUG repeats [66]. The screen identified the d-amino acid hexapeptide (ABP1) compound that is able to bind to CUG repeats, block their interaction with RNA biding proteins, leading to suppression of CUG-induced lethality and muscle degeneration. *In vitro* analysis demonstrated that ABP1 binds to CUG repeats with a high affinity and transforms double-stranded CUG RNA to single-stranded conformation, thus reducing CUG-RNA foci formation and Mbl aggregation. Importantly, the treatment of DM1 mouse model (expressing 250 CTG in the 3′UTR of the human skeletal actin open reading frame) by ABP1 rescues missplicing and improves muscle histopathology (Table 1). The conservation of the effect of ABP1 as a suppressor of RNA toxicity in both *Drosophila* and mouse DM1 models [66] suggests it could be tested to treat DM1 patients. 

More recently, Ligand 3, a new compound, has been reported as an alternative treatment strategy to reduce toxic foci formation (Table 1) [67]. This Ligand 3 is able to bind three consecutive CUG repeats instead of only one. *In vitro* and *in vivo* experiments confirmed its ability to reduce ribonuclear foci and partially rescue misregulated splicing of *cardiac troponin T* (*cTNT*) and *insulin receptor* (*IR*), two preRNAs mis-spliced in the DM1 context. More significantly, Ligand 3 partially rescued the degenerative phenotypes of DM1 flies [67]. Ligand 3 was later combined into a new bivalent ligand (Ligand 2a). In addition to inhibiting the MBNL1-CUG interaction *in vitro* and dissolving nuclear foci in DM1 cells, treatment with this substance ameliorated DM1 phenotypes in *Drosophila* including the adult external eye degeneration and larval crawling defect (Table 1) [68].

To identify potential therapeutic entities against DM1 cardiac dysfunction, Chakraborty and co-workers screened a drug library using a *Drosophila* model expressing 250 CTG repeats specifically in the heart. This led to the identification of pentamidine, which not only released Mbl from toxic foci in DM1 cardiomyocytes, but also rescued heart arrhythmicity and contractility and improved DM1 fly survival (Table 1) [46].

More recently, another drug, daunorubicin hydrochloride, was also found to bind to CUG repeats and inhibit Mbl sequestration (Table 1). Daunorubicin treatment resulted in the correction of Mbl-dependent splicing alterations and led to a better cardiac function recovery compared with pentamidine treatment [29].

All these examples thus support the DM1 fly model is an attractive system for identifying and testing genetic and substance-based DM1 treatment strategies.

## 6. Conclusion and Remarks

In this review, we have discussed how the application of the *Drosophila* model improves our understanding of the genetic and molecular bases of DM1 and helps identify therapeutic strategies. Although CTG repeat-toxicity is a principal factor that induces DM1 disease via MBNL1 sequestration and CELF1 stabilization, specific gene deregulations underlying different DM1-associated phenotypes remained poorly understood. *Drosophila* DM1 models have considerably accelerated the discovery of deregulated genes and pathways, including autophagy and apoptosis regulators, generation of siRNA from bidirectional transcription of long CTG repeats and aberrant expression of several miRNAs. The fly DM1 model has also proved to be well-adapted for genetic and chemical modifier screens for identifying new drugs able to reduce CTG toxicity *in vivo*. One important advantage of the *Drosophila* model is that it allows the use of the inducible GAL4/UAS system and thereby the analysis of DM1 pathogenesis in individually targeted organs and tissues. This was crucial for identifying muscle and heart-specific gene deregulations and also for selecting active substances rescuing DM1-associated heart phenotypes. 

Looking into the future, the recent advances in CRISPR/Cas9 genome editing systems offer a way to eliminate toxic repeat expansions or impede their transcription by deactivated Cas9. Here again, *Drosophila* offers an attractive model system for testing the efficacy of these new strategies.

## Figures and Tables

**Table 1:** Gene and drug screens in fly DM1 models.

Screen	Identified Drug	*Drosophila* Model	Phenotype/Mode of Action	Ref.
**Genetic Modifier Screening**	Suppressors: cnc, Nurf-38, foi, coro, csk, spinster; Enhancers: seven up, viking, cg4589	480 interrupted CTG	CUG-induced rough-eye phenotype	[37]
	Smaug	480 interrupted CTG	Restoration of translational activity of CELF1/Bru-3, rescue of CUG-induced myopathy (prevents muscle wasting/restore muscle function)	[60]
	apoptosis/autophagy inhibitors (overexpression of DIAP1, mTOR or muscleblind)	480 interrupted CTG	Rescue of the muscle-loss phenotype (atrophy)	[61]
**Drug Screening**	Non-steroidal anti-inflammatory agents, dopamine receptors and monoamine uptake inhibitors, Na^+^ and Ca^2+^ metabolism, Muscarinic, cholinergic and histamine receptors inhibitors	480 interrupted CTG)	Suppression of CUG-induced lethality	[37]
	d-amino acid hexapeptide (ABP1)	480 interrupted CTG	Biding to CUG repeats, elimination of CUG-RNA hairpin formation, suppression of CUG-induced lethality and muscle degeneration	[66]
	Ligand 3	480 interrupted CTG	Inhibition of MBNL1-CUG interaction, reduction of ribonuclear foci, partial rescue of misregulated splicing and degenerative phenotypes	[67]
	Ligand 2a	480 interrupted CTG	Inhibition of MBNL1-CUG interaction, reduction of ribonuclear foci, amelioration of adult external eye degeneration and larval crawling defect	[68]
	Pentamidine	250 pure CTG repeats	Inhibition of MBNL1-CUG interaction, reduction of ribonuclear foci, rescue of heart arrhythmicity and contractility, fly survival	[46]
	Daunorubicin hydrochloride	250 pure CTG repeats	Inhibition of MBNL1-CUG interaction, rescue of Mbl-dependent missplicing/cardiac function recovery (systolic interval and diastolic interval), fly survival	[29]
